# Four-dimensional vibrational spectroscopy for nanoscale mapping of phonon dispersion in BN nanotubes

**DOI:** 10.1038/s41467-021-21452-5

**Published:** 2021-02-19

**Authors:** Ruishi Qi, Ning Li, Jinlong Du, Ruochen Shi, Yang Huang, Xiaoxia Yang, Lei Liu, Zhi Xu, Qing Dai, Dapeng Yu, Peng Gao

**Affiliations:** 1 Electron Microscopy Laboratory, School of Physics, Peking University, Beijing, China; 2 International Center for Quantum Materials, Peking University, Beijing, China; 3 Department of Physics, University of California at Berkeley, Berkeley, CA USA; 4 Academy for Advanced Interdisciplinary Studies, Peking University, Beijing, China; 5 School of Materials Science and Engineering, Hebei University of Technology, Tianjin, China; 6 Hebei Key Laboratory of Boron Nitride Micro and Nano Materials, Hebei University of Technology, Tianjin, China; 7 CAS Key Laboratory of Nanophotonic Materials and Devices, CAS Center for Excellence in Nanoscience, National Center for Nanoscience and Technology, Beijing, China; 8 School of Materials Science and Engineering, Peking University, Beijing, China; 9 Songshan Lake Materials Lab, Institute of Physics, Chinese Academy of Sciences, Guangdong, China; 10 Shenzhen Key Laboratory of Quantum Science and Engineering, Shenzhen, China; 11 Collaborative Innovation Center of Quantum Matter, and Beijing Key Laboratory of Quantum Devices, Beijing, China

**Keywords:** Nanoscale materials, Techniques and instrumentation, Condensed-matter physics

## Abstract

Directly mapping local phonon dispersion in individual nanostructures can advance our understanding of their thermal, optical, and mechanical properties. However, this requires high detection sensitivity and combined spatial, energy and momentum resolutions, thus has been elusive. Here, we demonstrate a four-dimensional electron energy loss spectroscopy technique, and present position-dependent phonon dispersion measurements in individual boron nitride nanotubes. By scanning the electron beam in real space while monitoring both the energy loss and the momentum transfer, we are able to reveal position- and momentum-dependent lattice vibrations at nanometer scale. Our measurements show that the phonon dispersion of multi-walled nanotubes is locally close to hexagonal-boron nitride crystals. Interestingly, acoustic phonons are sensitive to defect scattering, while optical modes are insensitive to small voids. This work not only provides insights into vibrational properties of boron nitride nanotubes, but also demonstrates potential of the developed technique in nanoscale phonon dispersion measurements.

## Abstract (web summary)

Mapping local phonon dispersion in individual nanostructures reveals their thermal, optical, and mechanical properties but requires high detection sensitivity. Here, the authors present position-dependent phonon dispersion measurements in individual boron nitride nanotubes.

## Introduction

Phonon plays a fundamental role in mechanical, electrical, optical, and thermal properties of materials. There has been significant interest in measuring phonon dispersion in the hope of gaining mechanistic understandings and optimizing materials’ properties in materials science and condensed matter physics. However, such a measurement is very challenging for crystal defects, heterointerfaces and nanostructures, where the tiny size requires high spatial resolution and high detection sensitivity. Although the tip enhanced Raman spectroscopy and scanning near-field optical microscopy^1^ can reach nanometer spatial resolution, their momentum transfer is much smaller than the typical Brillouin zone (BZ) size and thus inaccessible to the high momentum phonons. Other vibrational spectroscopy techniques such as inelastic X-ray/neutron scattering are capable of measuring phonon dispersions for bulk crystals, but the lack of spatial resolution (limited by their beam size and low sensitivity) results in scattering signal averaged over large crystals or ensembles of nanostructures, hence precluding phonon dispersion measurements in individual nanostructures^2,3^.

Recent developments of aberration correctors and monochromators within in scanning transmission electron microscopes (STEMs) have enabled kiloelectronvolt electron beams with sub-10 meV energy resolution and atomic spatial resolution, extending electron energy loss spectroscopy (EELS) measurements into lattice vibration properties in the past decade^4,5^. Many spatially resolved measurements such as atomic-resolved phonon spectroscopy^6–9^, phonon polariton (PhP) mapping^10–12^, isotope identification^13^, and temperature measurement^14^ are now attainable. Recently, momentum-resolved vibrational measurements of hexagonal-boron nitride (h-BN) and graphite flakes using STEM-EELS have also been reported^15,16^. Although previous studies have demonstrated high sensitivity and large momentum transfer range of this technique, they only focused on flakes and two-dimensional (2D) sheets that are essentially homogeneous in space, and thus did not take advantage of the high spatial resolution of electron microscopes. Long acquisition time (up to ~10 h for each dispersion diagram at a single spatial location) of the serial acquisition method^15^ also precludes the possibility to perform a 2D scan or even a line scan. To date, nanoscale position-dependent phonon dispersion measurement in a single nanostructure has not been reported to the best of our knowledge.

Here, we report an efficient acquisition method for real-space mapping of phonon dispersions in a single nanostructure. With a slot aperture, phonon dispersion data can be acquired in parallel within much shorter acquisition time^17^. This allows us to add two spatial dimensions to this technique and enables four-dimensional EELS (4D-EELS) measurements in a single nanostructure at nanometer scale. As a model system, boron nitride nanotubes (BNNTs) have shown remarkable mechanical, thermal, and nano-optical properties that promise a wide range of applications, such as heat conductors for nanoelectronics and low-loss polaritonic devices^18–23^. These properties are dominated by lattice vibrations because the electronic contributions are nearly negligible due to a large bandgap. The knowledge about their phonon properties is therefore highly desired. Ab initio and zone-folding calculations have predicted an unfolded phonon dispersion of BNNTs similar to that of two-dimensional h-BN layers^24^, but experimental verification has been absent. In addition, in multi-walled BNNTs interlayer coupling usually lead to vacancies, faceting, and change of stacking order^18,25–28^, whose impacts on phonon properties still await experimental exploration. Unfortunately, traditional spectroscopy techniques cannot measure their phonon dispersion even for ensembles of BNNTs, not to mention for individual ones. This is because their cylindrical shape results in a crystal orientation varying with the azimuthal angle, which means broad-beam scattering scheme will inevitably mix up scattering signals with momenta along all directions; nonuniform chirality and diameter distribution among BNNTs will also mix up phonon signals from BNNTs with different structures and/or orientations. With fine spatial resolution substantially smaller than the tube diameter, the 4D-EELS technique is evidently ideal to probe their local vibration properties.

Our measurements reveal that the unfolded phonon dispersion of multi-walled BNNTs is very similar to that of h-BN crystals, which indicates curved geometry of atom planes and interlayer coupling have no substantial impact on the phonon dispersion. Real-space mapping of different phonon modes at various momentum transfers shows that acoustic phonon modes in BNNTs are susceptible to defect scattering, while high-frequency optical modes are less sensitive to small voids. These results provide useful information about phonons and associated properties of BNNTs, and demonstrate new possibilities in probing local phonon structures.

## Results

### Phonon measurements in a STEM

**Fig. 1:**
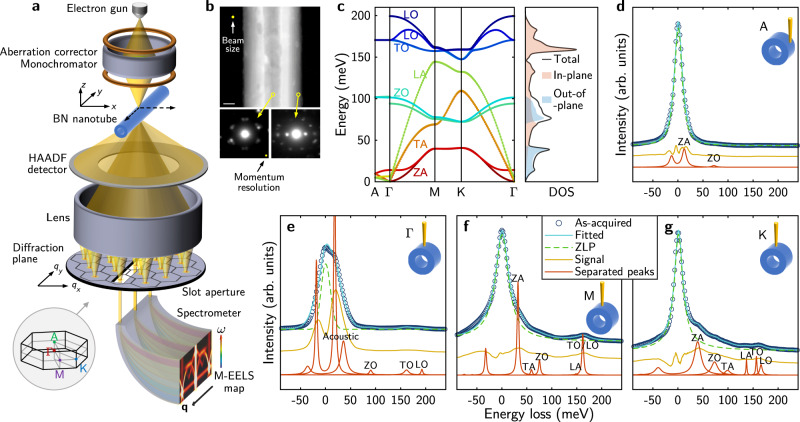
4D-EELS in a STEM. **a** Schematic of the experimental setup. The inset at the bottom shows high-symmetry points in the first BZ of h-BN. **b** A typical HAADF image of a BNNT (top panel). Scale bar, 20 nm. Two bottom panels are electron diffraction patterns with the beam located at the center (left) and near the edge (right) of the tube. **c** Phonon dispersion curve and phonon DOS of h-BN crystal calculated by DFPT. Light pink and light blue shadows are in-plane and out-of-plane contributions. **d**–**g** Typical EEL spectra at high-symmetry points A, Γ, M, and K in the unfolded BZ of a BNNT. Dark-blue circles represent the raw spectra, to which solid cyan lines are fitted (Methods). Dashed green lines are quasi-elastic lines extracted from the fitting, which are subtracted from the measured spectra to get phonon signal (yellow lines). Red curves are separated multiple phonon peaks, the summation of which, once convoluted with the fitted quasi-elastic line, gives the least difference with the measured signal. For clarity lines are vertically shifted.

The experimental setup is illustrated in Fig. 1a. A kiloelectronvolt electron beam is focused to nanometer-wide, with its spatial and energy resolutions further refined by the aberration correctors and the monochromator. After scattering from the sample, electrons pass through magnetic lenses to form diffraction spots on the diffraction plane, where an EELS aperture is placed to select scattered electrons with particular momentum transfers. Previous momentum-resolved EELS (M-EELS) studies collect phonon dispersion data serially by moving a small round entrance aperture relative to the diffraction plane^15,16^. A more efficient way for M-EELS data acquisition, which reduces the typical acquisition time for each dispersion diagram from several hours to tens of minutes, is to use a slot aperture to select a narrow line on the diffraction plane^12,29,30^, producing a 2D intensity map versus both energy and momentum transfer. This scheme is also advantageous for data consistency among momentum points (avoiding beam instabilities over time) and for a better momentum resolution with a much higher momentum sampling density. With the beam scanning in two spatial dimensions, a 4D dataset is recorded. The sample morphology is also recorded using high-angle scattered electrons at the same time, giving us information about the microstructure of BNNTs. Top panel of Fig. 1b is a high-angle annular dark field (HAADF) image of a typical BNNT, from which the inner and outer radius can be easily determined. From the electron diffraction patterns shown in the bottom panel, its chirality can be determined close to zigzag. This allows us to characterize the structure of individual BNNTs under investigation. Although the spatial resolution and momentum resolution can be separately optimized to 0.1 nm and <0.1 Å^−1^ by choosing a very large and a very small convergence semi-angle respectively, there is an intrinsic tradeoff between them. Here, after balancing the spatial and momentum resolutions the typical beam size is estimated to be ~4 nm while keeping a momentum resolution of ~0.3 Å^−1^ (Supplementary Fig. 1).

Figure 1d–g shows typical EEL spectra at high-symmetry points of the unfolded BZ, A, Γ, M, and K respectively (see inset of Fig. 1a). Apart from a strong quasi-elastic line located at zero energy (zero loss peak, ZLP), in the range from ~10 to 200 meV we observe multiple phonon modes with their intensity and energy varying with the momentum transfer. Because of the worse energy resolution and large variation of phonon peak energy and intensity in M-EELS maps, removal of the quasi-elastic line (background subtraction) is not as straightforward as in conventional EELS. Here we develop a fitting method (Methods) to remove the quasi-elastic line (green dashed lines in Fig. 1d–g) and simultaneously separate phonon peaks (red traces). At Γ point (Fig. 1e), acoustic modes have small but nonzero frequencies due to their large group velocity and our finite momentum resolution. The phonon occupation number diverges exponentially near zero frequency, resulting in a very strong intensity for acoustic modes. Since they are close in energy, it is hard to fully separate them and assign them to specific modes. Except these two peaks, all other phonon peaks can be assigned to six types (ZA, TA, LA, ZO, TO, and LO) by comparing with the calculated phonon dispersion. Figure 1c shows the density functional perturbation theory (DFPT) calculation of phonon dispersion and phonon density of states (DOS) for a bulk h-BN crystal. Phonon peaks in the experimental data matches reasonably well with the calculation.

Apart from the energy loss peaks, we also observe energy gain peaks on the left side of the ZLP. This corresponds to an induced phonon absorption event of the electron beam^31^. Assuming detailed balance, the intensity ratio between the energy gain peak and the energy loss peak is the Boltzmann factor $\exp \left( { - \frac{{\hbar \omega }}{{k_{\mathrm{B}}T}}} \right)$, where *ħ* is the reduced Planck constant, *ω* is phonon frequency, *k*_B_ is the Boltzmann constant and *T* denotes the temperature (~293 K in our experiment)^14^. At room temperature this is observable for low-frequency acoustic modes, but decays quickly with higher energy and becomes undetectable for optical modes.

### 4D-EELS in individual BNNTs

**Fig. 2:**
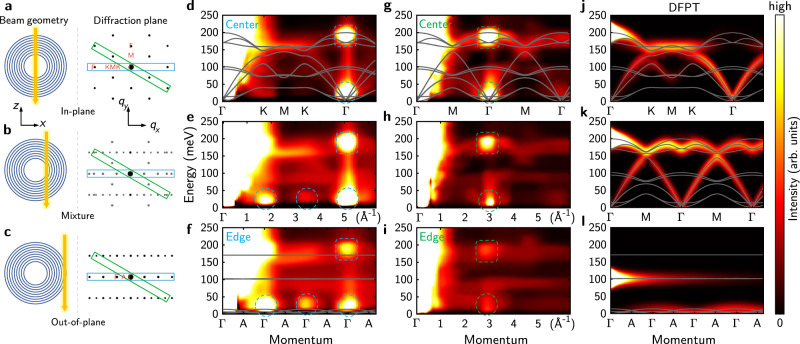
4D-EELS measurement of phonon dispersion in an individual zigzag BNNT. **a**–**c** Schematic of the experimental geometry. Left panels illustrate the beam position (yellow arrows). Right panels show the diffraction plane. Blue and green rectangles illustrate the position of the slot aperture used in **d**–**f** (Supplementary Movie 1) and **g**–**i** (Supplementary Movie 2), respectively. The underlying black dot patterns are simulated electron diffraction patterns, in which the vertical direction is parallel to the tube axis. **d**–**f** Phonon dispersion line profiles along a radius of the BNNT, acquired with the slot aperture placed along the blue rectangle in **a**–**c**. **d**, **f** correspond to the tube center and the edge respectively, and **e** is acquired between them. Dashed circles and squares mark bright spots from Bragg diffraction and multiple scattering processes. **g**–**i** Same as **d**–**f**, except that the aperture is long the green rectangle in **a**–**c**. **j**–**l** Calculated EELS intensity (statistical factor corrected) for a bulk h-BN crystal along high-symmetry lines ΓKMKΓ (**j**), ΓMΓMΓ (**k**), and ΓAΓAΓ (**l**). Gray curves are calculated dispersion for bulk h-BN crystals.

To visualize spatial-dependent phonon dispersion in a single BNNT, we performed two 4D-EELS measurements for a zigzag BNNT. Supplementary Fig. 2 shows the HAADF image and electron diffraction patterns of the BNNT under investigation. Supplementary Movies 1 and 2 show two 4D datasets acquired with the slot aperture placed in two directions (schematically shown in Fig. 2a–c), in which the local phonon dispersion substantially changes with position. In contrast, for a h-BN flake with uniform thickness, the phonon dispersion remains the same while scanning on the sample (Supplementary Movies 3 and 4, and Supplementary Fig. 3). As a concise summary of the 4D datasets, Fig. 2d–i show typical dispersion diagrams with the beam scanning from the tube center (Fig. 2a, d, g) to the edge (Fig. 2c, f, i).

The position-dependent dispersion diagrams can be well captured by viewing the nanotube locally as h-BN flakes rotated by a spatial-dependent tilt angle. When the beam passes through the center of the BNNT (Fig. 2a, d, g), the configuration is similar to the case of two vertically stacked h-BN flakes, with their relative rotation angle determined by the chiral angle of the tube. For zigzag BNNTs the two flakes are rotationally aligned, so in-plane high-symmetry points Γ, M, and K are well-defined for scattered electrons. Measured phonon dispersion diagrams along ΓKMKΓ and ΓMΓMΓ lines are displayed in Fig. 2d, g, respectively. As a reference, we also performed measurements for a h-BN flake under similar experimental conditions, with the results shown in Supplementary Fig. 4. As expected, they share common overall features as a manifestation of their similar local structures. To understand and corroborate these measured spectra, Fig. 2j–l display calculated EEL spectra for bulk h-BN crystals along high-symmetry lines based on DFPT and scattering cross section formalism^15,32^ (Methods). Because the scattering cross section is proportional to the inner product between the momentum transfer **q** and the displacement vector of the *k*^th^ atom and *λ*^th^ mode **e**_*λ*_(*k*,**q**), only modes with nonzero vibration displacement along **q** will be active in EELS measurements^32^. For h-BN flakes with normal electron beam incidence, ZO and ZA modes are not active since they have displacement vectors normal to atom planes, which are always perpendicular to the in-plane momentum transfer **q**. Nice agreement is found between the experiment and the calculated spectra.

At the tube edge (Fig. 2c, f, i), the geometry is similar to a h-BN flake tilted along an armchair direction by 90°. Therefore, in Fig. 2f we get the out-of-plane dispersion along ΓAΓAΓ line, where the phonon dispersion is highly nondispersive due to the weak van der Waals (vdW) interlayer coupling.

Between the tube center and the tube edge, the electron beam passes through multiple shells with different local crystal orientation (Fig. 2b, e, h). Due to the curved geometry, outer shells will have more in-plane contribution. Thus, the scattering signal is a mixture projected along the beam trajectory. Approaching the edge, the average tilt angle increases and the out-of-plane contribution becomes larger, so the dispersion gradually flattens, which is clearly observable in Supplementary Movies 1 and 2. We observe that the intensity of ZO and ZA modes increases from the tube center to the edge, due to an increasing inner product between **q** and **e**_*λ*_(*k*,**q**); meanwhile, the signal of in-plane modes becomes weaker. This is in qualitative agreement with scattering cross section theory. However, the ZO and ZA modes still have a small but nonzero intensity at the tube center, and the TO/LO mode also remains considerable intensity at the tube edge. This may be due to the finite probe size and the curved geometry of atom shells, which slightly alters the vibration direction of each atom and leads to nonzero value of **q** · **e**_*λ*_(*k*,**q**).

We noticed that the collected dispersion diagrams contain some bright spots (dashed circles and squares in Fig. 2d–i). They do not indicate abnormal behaviors of lattice vibrations at corresponding momenta; instead, they come from strong Bragg diffraction and associated multiple scattering processes (see Supplementary Fig. 2b–j for electron diffraction patterns). The enhancing of signal near TO/LO energy (dashed squares) is mainly from multiple scattering, such as one (or more) elastic scattering with momentum transfer equal to a reciprocal lattice vector followed by an inelastic phonon scattering with a negligible momentum transfer. At small **q**, the phonon scattering cross section diverges as 1/*q*^2^ (Methods), and dipole scattering is also strong, so the collected signal at Bragg spots is stronger than nearby momenta. At low energy (dashed circles), apart from multiple scattering, a small residual of the strong elastic line during background subtraction may also introduce fictitious bright spots in the dispersion diagram.

**Fig. 3:**
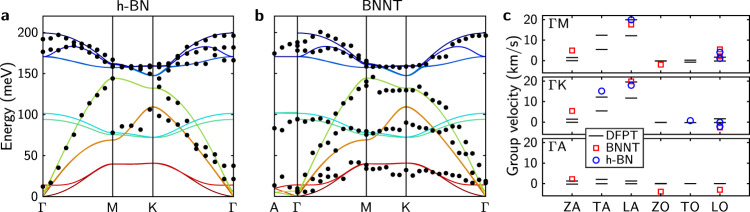
Phonon dispersion and group velocity. **a**, **b** Phonon dispersion of the h-BN flake and the BNNT along high-symmetry lines. Extracted energies are averaged among multiple BZs and shown as black dots. Solid curves are DFPT calculation for bulk h-BN crystals. **c** Anisotropic phonon group velocity near Γ point. Red and blue scatters are extracted from the experimental data by fitting a quadratic function near the Γ point. Black lines are derivatives of the DFPT calculated dispersion.

By averaging among multiple BZs, we extract unfolded phonon dispersion of the BNNT and the h-BN flake from the measured spectra, as displayed in Fig. 3. They both match well with the DFPT calculation, and their difference is beyond our energy resolution. The similarity in phonon dispersion of h-BN crystals and BNNTs implies that effects of structural curvature and interlayer coupling on phonon dispersion are insignificant. First, phonon dispersion in vdW crystals is insensitive to the shape of the atom plane. In fact, the zone-folding method for numerical study of nanotubes simply constructs phonon dispersion (or band structure) of nanotubes from that of a corresponding sheet with a periodic boundary condition imposed^24,33^. The underlying assumption of this scheme is that the effect of shell curvature is negligible, and this is supported by our observation at least for BNNTs with a radius of several tens of nanometers. Ab initio calculations have also shown that this method works very well for single-walled BNNTs^24^ and carbon nanotubes^34^ with smaller radius. Second, their phonon dispersion depends weakly on the vdW interlayer interaction. Due to inevitably incommensurate perimeters between neighboring shells, the chiral angle, stacking order, and interlayer distance may vary^25,35,36^, so the interlayer coupling in BNNTs can be different from that of a bulk h-BN crystal. Supplementary Fig. 5 compares calculated phonon dispersions for AA’ and AB staking bulk crystals, whose similarity indicates weak vdW interlayer coupling does not substantially affect the phonon dispersion, in agreement with our measurements.

From the phonon dispersion data, we extract phonon group velocity near the Γ point along three directions (Fig. 3c). While two in-plane directions ΓM and ΓK share similar group velocities, in the out-of-plane direction the group velocity is almost an order of magnitude smaller. This suggests the thermal conductivity in BNNTs is highly anisotropic, with the radial component much smaller than that along the axis and the azimuthal direction.

### Momentum- and mode- dependent defect scattering

**Fig. 4:**
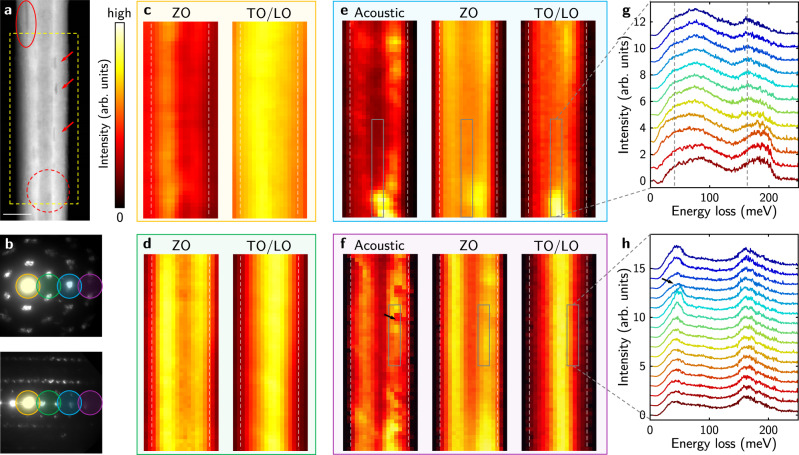
Real-space mapping of phonon signal at various momentum transfers. **a** HAADF image of a near-zigzag BNNT, in which structural defects can be clearly observed. Yellow dashed box encircles the scanning region in **c**–**f**. Scale bar, 50 nm. **b** Electron diffraction patterns taken near the center (top panel) and the edge (bottom panel) of the BNNT. Colored circles denote four selected momentum transfers that correspond to **c**–**f**. The diffraction peak with highest intensity (encircled by the yellow circle) is the central diffraction spot with zero momentum transfer. The momentum transfer increases with a step of ~2.5 Å^−1^ from **c** to **f**. **c**–**f** Intensity maps of the acoustic, ZO (possibly with some LA signal) and TO/LO phonon modes in real space. At small momentum transfers (**c**, **d**), the quasi-elastic line dominates the low-energy region, so the acoustic phonon signal is hard to extract. **g** EELS line profile (after background-subtraction and statistical factor correction) along the gray rectangle in **e**. Two vertical dashed lines are a guide to the eye. **h** EELS line profile along the gray rectangle in **f**. Black arrow marks the position of the void. In **g**, **h**, each line is successively shifted upward for clarity. For completeness, the raw spectra without background-subtraction are shown in Supplementary Fig. 7.

Note that due to large strain inherent in their structure, defects are common in multi-walled BNNTs^25^. With fine spatial resolution, we can actually map the intensity distribution of these phonon modes in real space and reveal the influence of defects. Phonon intensity maps for a high-quality h-BN flake (Supplementary Fig. 3) confirm that when defect scattering is absent, the phonon signals are homogeneous in space. Shown in Fig. 4a is a HAADF image of a BNNT with obvious structural irregularities. On the top-left corner, the red ellipse marks a dark line in the image that extends far beyond the top boundary of this figure, which corresponds to a gap between atom shells in this BNNT. Red arrows highlight voids in some constituting shells (see Supplementary Fig. 6 for the atomic structure of a typical void). We select four momentum transfers (illustrated in Fig. 4b) to spatially map the phonon signal. Starting from the central diffraction spot, the momentum increases with a step size of ~2.5 Å^−1^ perpendicular to the tube axis. At (nearly) zero momentum transfer, the electron beam interacts with lattice vibration of BNNTs predominately through dipole scattering, which essentially yields PhP signals^12,37^. In Fig. 4c, the PhP signal extends outside the tube (outlined by white dashed lines), and is not very sensitive to small defects. This is because the long-range Coulomb interaction is on a length scale comparable to the tube radius, which smears out small local variations. However, since the PhPs only have very small momentum compared to the BZ size, the vibrational signal will be increasingly localized with larger momentum transfer. At the first BZ boundary (Fig. 4d) the spectra mainly contain impact scattering signal that is localized within the BNNT. The ZO mode is more intense near the edge, while TO/LO signal is stronger near the center, in agreement with the **q** · **e**_*λ*_(*k*,**q**) dependence of the scattering cross section. At even higher momentum transfers, the quasi-elastic line is weaker so extracting acoustic phonon signal is possible. In Fig. 4e, f, the acoustic phonon maps show apparent correlation with the structral defects observed in the HAADF image, and the contrast is larger for higher momentum transfer. On the other hand, although optical branches are also slightly affected by the presence of voids, the contrast is much weaker. This is also confirmed by the EELS line profile shown in Fig. 4h across a void, where the acoustic phonon signal decreases significantly on the defect while the optical modes remain almost the same. Being sensitive to defect scattering, phonon intensity maps, especially acoustic ones, may be used to identify defects that are hard to visualize by conventional imaging techniques. This also means defects play an important role in thermal transport in BNNTs as the acoustic phonons contribute a vast majority of thermal conductivity because of their higher group velocities^38^. The presence of defects will reduce their lifetimes and hence substantially reduce the thermal conductivity.

Interestingly, a strong hot spot appears at the bottom of these phonon intensity maps, while in this region no obvious contrast can be seen in the HAADF image (dashed circle in Fig. 4a). This defect shows largest contrast in phonon maps at medium momentum transfers (Fig. 4e), and unlike voids, it also considerably changes optical phonon signal. This is unexpected since optical phonons have been reported to be sensitive only to thickness but insensitive to small scale defects^15^. To further verify that this hot spot is not from artifects such as diffraction effects or beam intensity fluctuation, Fig. 4g presents an EELS line profile across this defect region. Scattering intensity for both acoustic and TO/LO modes is significantly increased on this defect, accompanied by an energy shift. The center of the TO/LO peak blueshifts from ~165 to ~185 meV, while the acoustic peak slightly redshifts. Further theoretical and experimental studies are needed in future to fully understand these scattering behaviors, and to verify whether this is due to the emergence of a new defect mode, or a scattering intensity change caused by a local structure variation.

## Discussion

We have experimentally measured local phonon dispersion relations in individual BNNTs to reveal both position-dependent and momentum-dependent lattice vibration behaviors using the 4D-EELS technique. We show that although the phonon dispersion in BNNTs is position-dependent, the main features are similar to that of bulk h-BN crystals. Real-space mapping emphasizes the importance of defect scattering for understanding acoustic phonons and related material properties such as thermal transport. These conclusions should probably apply to many other similar nanostructures such as carbon nanotubes. We have to admit, though, that more detailed measurements are still limited by the energy resolution. Currently, the uncertainty of extracted dispersion curves mainly comes from (i) nonideal monochromaticity of the electron beam, which smears out spectrum details and mixes up peaks close to each other; (ii) limited momentum resolution (especially when high spatial resolution is required), which broadens phonon peaks, especially acoustic ones, due to a smearing in the momentum space; and (iii) challenges in post-processing methods and data interpretation. Further improvement will require efforts in both equipment hardware and algorithms. With higher resolution, the small dispersion change associated with interlayer coupling and defects may become detectable, then 4D-EELS could easily correlate phonon dispersion with small structural changes such as chiral angle, local stacking order, and tiny defects. Nevertheless, we anticipate that the demonstrated 4D-EELS technique will surely find more applications in vibrational measurements of many other interesting material systems, such as resolving defect phonon modes^39^, interface phonon modes, and even topological phonon surface/edge states^40^.

## Methods

### EELS data acquisition

The EELS data was acquired on a Nion U-HERMES200 microscope equipped with both a monochromator and aberration correctors, with ~12 pA beam current. A relatively small beam convergence semi-angle of 1.5 mrad was used for a balanced momentum and spatial resolution, and the collection angle of the slot aperture was 4.5  × 75 mrad. Two BNNT 4D datasets (Supplementary Movies 1 and 2) were acquired under 60 keV beam energy with a total integration time of 36 min for each dataset (12 pixel × 18 pixel × 10 s). Under such conditions, the typical spatial, momentum, and energy resolutions are 4 nm (estimated by comparing the acquired HAADF image to an ideal one), 0.3 Å^−1^ (along the slot, estimated by the diffraction spot size) and 15 meV (FWHM of the quasi-elastic line), respectively (see Supplementary Fig. 1). Two h-BN 4D datasets (Supplementary Movies 3 and 4) were acquired under 30 keV beam energy with a total integration time of 42 min for each dataset (25 pixel × 10 pixel × 10 s). Lower acceleration voltage gives a better energy resolution, but sacrifices spatial resolution and penetration depth (and hence lower signal for thick samples). This is suitable for data acquisition on h-BN flakes which does not require a very high spatial resolution or penetration depth (due to smaller thickness). However, for comparison the h-BN data was acquired again (Fig. 3a, Supplementary Fig. 4) under the same experimental conditions as those used for BNNT. Supplementary Table 1 gives detailed experimental parameters of these datasets.

### EELS data processing

All acquired spectra were processed by custom-written Matlab code. For each 4D-EELS dataset, EEL spectra were first registered by their normalized cross correlation to correct possible beam energy drift and momentum distortion. After the alignment, we applied block-matching and 3D filtering (BM3D) algorithm to remove Gaussian noise^41,42^. For each momentum and each energy channel, the data is denoised in two spatial dimensions, where the noise level is individually estimated based on high-frequency elements in the Fourier domain. A correction for the statistical factor is performed following literature^31^.

### Quasi-elastic line fitting

In vibrational EELS, the quasi-elastic line forms a strong ZLP in the measured spectra, the removal of which is usually not straightforward. For M-EELS data the energy loss peaks are at different energy for different momentum, making the commonly-used two-window method hard to perform due to difficulties in finding a common energy window. Besides, acoustic phonons at very low energy cannot be well separated from the rapidly-varying background by this method. As shown in Fig. 1, here the quasi-elastic line is removed by least-square fitting the measured spectrum (over the whole energy range, not only in some energy windows) to the following fitting function

1 $$P^\prime \left( \omega \right) + \mathop {\sum }\limits_\lambda a_\lambda \left[ {L\left( {\frac{{\omega - \omega _\lambda }}{{c_\lambda }}} \right) + \exp \left( { - \frac{{\hslash \omega _\lambda }}{{k_{\mathrm{B}}T}}} \right)L\left( {\frac{{\omega + \omega _\lambda }}{{c_\lambda }}} \right)} \right] * P^\prime \left( \omega \right)$$

where the first term is a modified Pearson-VII function^8,43^ to account for the quasi-elastic line, and the second term is the contribution of multiple phonon modes in which *L*(*x*) is the Lorentzian function. The asterisk symbol denotes convolution. The original Pearson-VII function is generally a Lorentzian raised by power *m*

2 $$P\left( \omega \right) = I_{\max }\frac{{\gamma ^{2m}}}{{\left[ {\gamma ^2 + \left( {2^{1/m} - 1} \right)\left( {2\omega - 2\omega _0} \right)^2} \right]^m}}$$

in which *I*_max_, *ω*_0_ and *γ* are the height, center and width of the Pearson peak. A modification (denoted by the prime symbol) is then made to account for possible ZLP asymmetry, where the constant width parameter *γ* is replaced by a function $\gamma \left( \omega \right) = \frac{{2\gamma _0}}{{1 + \exp \left( {s\omega } \right)}}$ containing an additional fitting parameter *s*. With these 5 fitting parameters, the ZLP can be fitted rather well. The inelastic scattering signal are modeled by paired Lorentzian peaks with central energy $\pm \omega _{\lambda}$, peak height *a*_*λ*_ and $a_\lambda \exp \left( { - \frac{{\hslash \omega _\lambda }}{{k_{\mathrm{B}}T}}} \right)$, and peak width *c*_*λ*_. These Lorentzian peaks are convoluted with the quasi-elastic line to account for the electron energy distribution. Each measured spectrum is fitted to the sum of these two contributions (Eq. 1), and then the fitted ZLP is subtracted from the measured spectrum to get the signal. In the fitting, the calculated phonon energy was used as the initial guess. For most measured spectra, the fitting converges to the same minimum within a reasonable range of initial guess, and typical relative fitting residual is ~5% (mainly from the low-energy region). A small portion of mis-identified peaks are manually removed.

### DFPT calculation

The DFPT calculation was performed using local density approximation and norm-conserving pseudopotentials with a 770 eV energy cutoff.

The scattering cross section for an infinite bulk crystal is^11,15,32^

3 $$\frac{{{\mathrm{{d}}}^2\sigma }}{{{\mathrm{{d}}}\omega {\mathrm{{d}}}\Omega }} \propto \mathop {\sum }\limits_{{\mathrm{mode}}\;\lambda } |F_\lambda \left( {\mathbf{q}} \right)|^2\left[ {\frac{{n_{\mathbf{q}} + 1}}{{\omega _\lambda \left( {\mathbf{q}} \right)}}\delta \left( {\omega - \omega _\lambda \left( {\mathbf{q}} \right)} \right) + \frac{{n_{\mathbf{q}}}}{{\omega _\lambda \left( {\mathbf{q}} \right)}}\delta \left( {\omega + \omega _\lambda \left( {\mathbf{q}} \right)} \right)} \right]$$

where *ω*_*λ*_(**q**) and *n*_**q**_ are the frequency and occupancy number of the *λ*^th^ phonon mode with wavevector **q**. The two terms in the square brackets correspond to phonon emission and absorption process respectively. The coupling factor

4 $$F_\lambda \left( {\mathbf{q}} \right) \propto \frac{1}{{q^2}}\mathop {\sum }\limits_{{\mathrm{atom}}\;k} \frac{1}{{\sqrt {M_k} }}e^{ - i{\mathbf{q}} \cdot {\mathbf{r}}_k}e^{ - W_k\left( {\mathbf{q}} \right)}Z_k\left( {\mathbf{q}} \right)\left[ {{\mathbf{e}}_\lambda \left( {k,{\mathbf{q}}} \right) \cdot {\mathbf{q}}} \right]$$

is determined by the mass *M*_*k*_, real-space position **r**_*k*_, effective charge *Z*_*k*_(**q**), Debye–Waller factor^44^ $e^{ - 2W_k\left( {\mathbf{q}} \right)}$ and phonon displacement vector **e**_*λ*_(*k*,**q**) of *k*^th^ atom in a unit cell. The effective charge *Z*_*k*_(**q**) was calculated using Eq.(9) in literature^32^ with atomic form factors of B and N constructed from parameters in literature^45^. After correction for the statistical factor, the experimental diagram is essentially proportional to $\mathop {\sum }\limits_\lambda |F_\lambda \left( {\mathbf{q}} \right)|^2$, so the DFPT calculated diagrams in the main text also show this quantity (convoluted with a Gaussian to broaden the delta function for better visibility).

## Supplementary information

Supplementary Information

Description of Additional Supplementary Files

Supplementary Movie 1

Supplementary Movie 2

Supplementary Movie 3

Supplementary Movie 4

## Data Availability

The data that support the findings of this study are available from the corresponding author upon request.

## Code availability

A GUI version of the matlab code for 4D-EELS data processing is available from the corresponding author upon request.
