# Supplementary material for: Four-dimensional vibrational spectroscopy for nanoscale mapping of phonon dispersion in BN nanotubes
Source: Nat Commun. 2021 Feb 19;12:1179. doi: 10.1038/s41467-021-21452-5 (PMC7896073; doi:10.1038/s41467-021-21452-5)
Supplement: Supplementary file 1 — Supplementary Information [file 41467_2021_21452_MOESM1_ESM.pdf]

Supplementary Information for

Four-dimensional Vibrational Spectroscopy for Nanoscale Mapping of Phonon Dispersion in BN Nanotubes

Ruishi Qi[1,2,3#], Ning Li[1,2,4#], Jinlong Du[1], Ruochen Shi[1,2], Yang Huang[5,6], Xiaoxia Yang[7], Lei Liu[8], Zhi Xu[9], Qing Dai[7], Dapeng Yu[10], and Peng Gao[1,2,11*]

1 Electron Microscopy Laboratory, School of Physics, Peking University, Beijing, China.

2 International Center for Quantum Materials, Peking University, Beijing, China.

3 Department of Physics, University of California at Berkeley, Berkeley, CA, USA.

4 Academy for Advanced Interdisciplinary Studies, Peking University, Beijing, China.

5 School of Materials Science and Engineering, Hebei University of Technology, Tianjin, China.

6 Hebei Key Laboratory of Boron Nitride Micro and Nano Materials, Hebei University of Technology, Tianjin, China.

7 CAS Key Laboratory of Nanophotonic Materials and Devices, CAS Center for Excellence in Nanoscience, National Center for Nanoscience and Technology, Beijing, China.

8 School of Materials Science and Engineering, Peking University, Beijing, China.

9 Songshan Lake Materials Lab, Institute of Physics, Chinese Academy of Sciences, Guangdong, China.

10 Shenzhen Key Laboratory of Quantum Science and Engineering, Shenzhen, China.

11 Collaborative Innovation Center of Quantum Matter, and Beijing Key Laboratory of Quantum Devices, Beijing, China.

# These authors contribute equally to this work: Ruishi Qi, Ning Li.

* Corresponding author. E-mail: p-gao@pku.edu.cn.

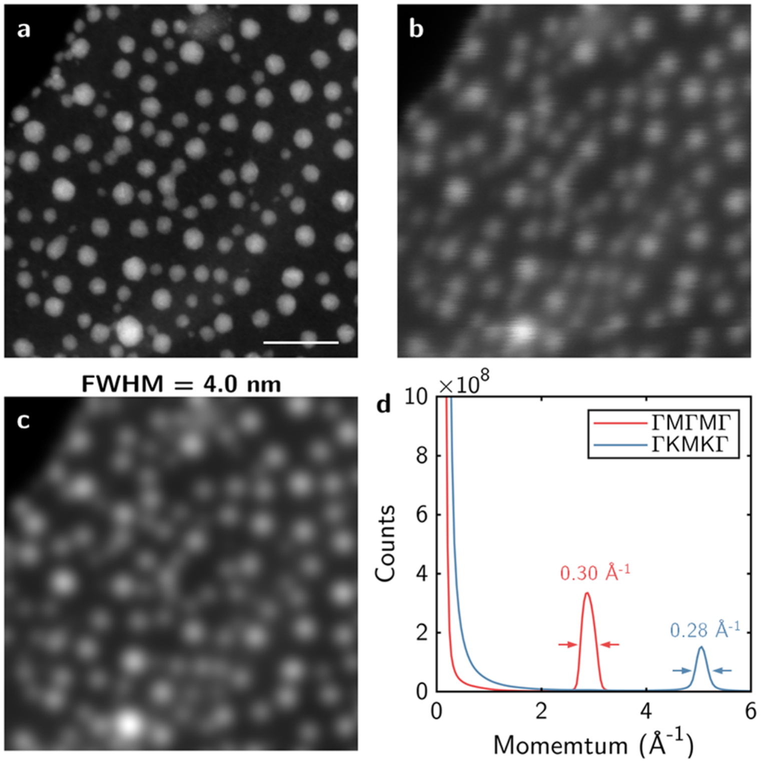

Supplementary Figure 1 Resolution estimation at 1.5 mrad convergence semi-angle and 60 keV beam energy. **a** HAADF image of gold nanoparticles taken with 20 mrad convergence semi-angle. The spatial resolution under such condition is known to be < 0.2 nm, so this image can serve as a reference. Scale bar, 20 nm. **b** HAADF image taken with 1.5 mrad convergence semi-angle (used in our 4D-EELS measurements). **c** The 20 mrad image convoluted with a Gaussian kernel, whose width is least-square fitted to achieve the best match between the convoluted image and the 1.5 mrad image. The fitted value of its FWHM is 4.0 nm. **d** Momentum resolution estimation. Solid curves show total EELS counts (summed over all energy channels) as a function of the momentum transfer. Red and blue curves are acquired on a high-quality h-BN flake along ΓMΓMΓ and ΓKMKΓ lines, respectively. The FWHM of the Bragg reflection spots can be used to estimate our momentum resolution along the slot, which is ~0.3 $Å^{-1}$ for both datasets. For the direction perpendicular to the slot, the slot half-width causes an additional smearing of 0.29 $Å^{-1}$.

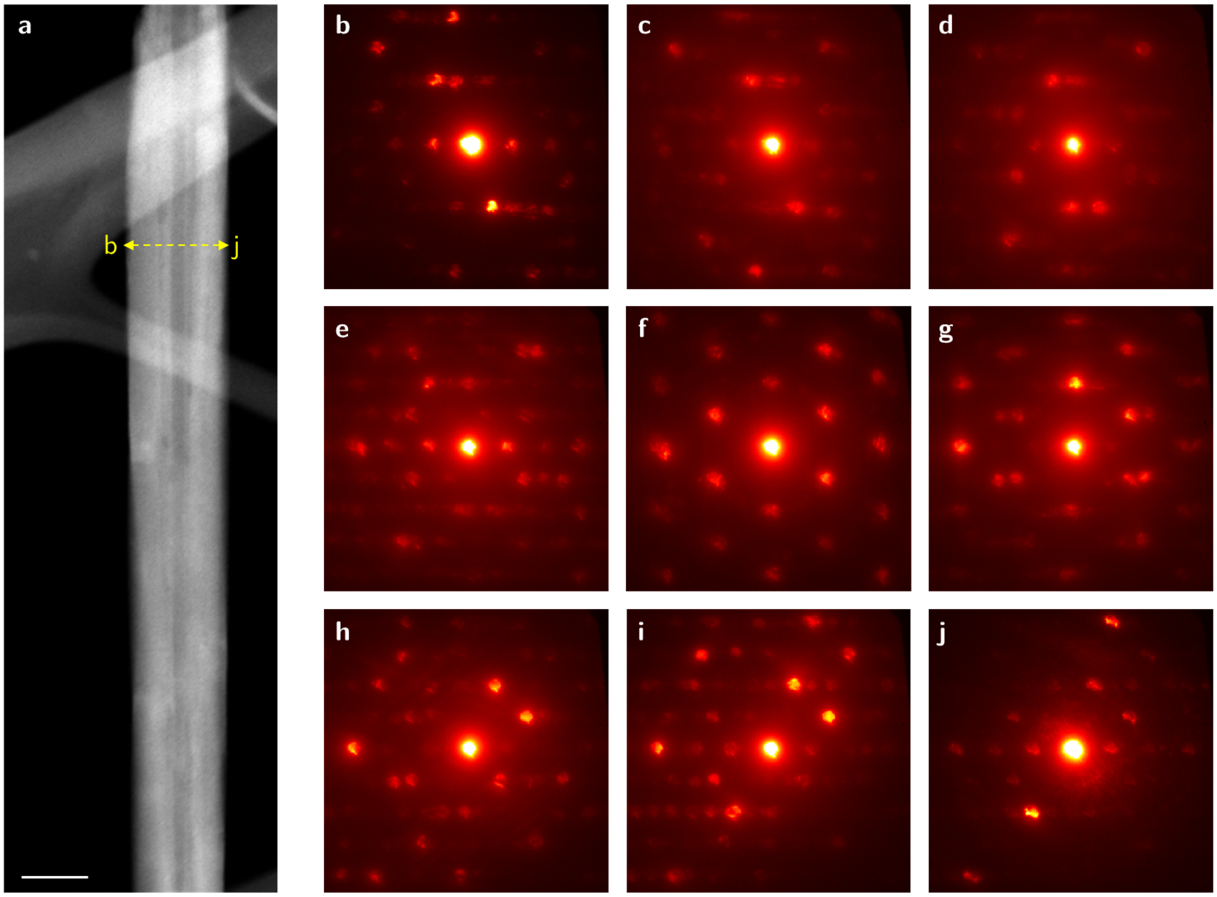

Supplementary Figure 2 HAADF image (**a**) and electron diffraction patterns (**b-j**) across a diameter of the BNNT (yellow dashed arrow) under investigation in Fig. 2. Scale bar in **a**, 100 nm.

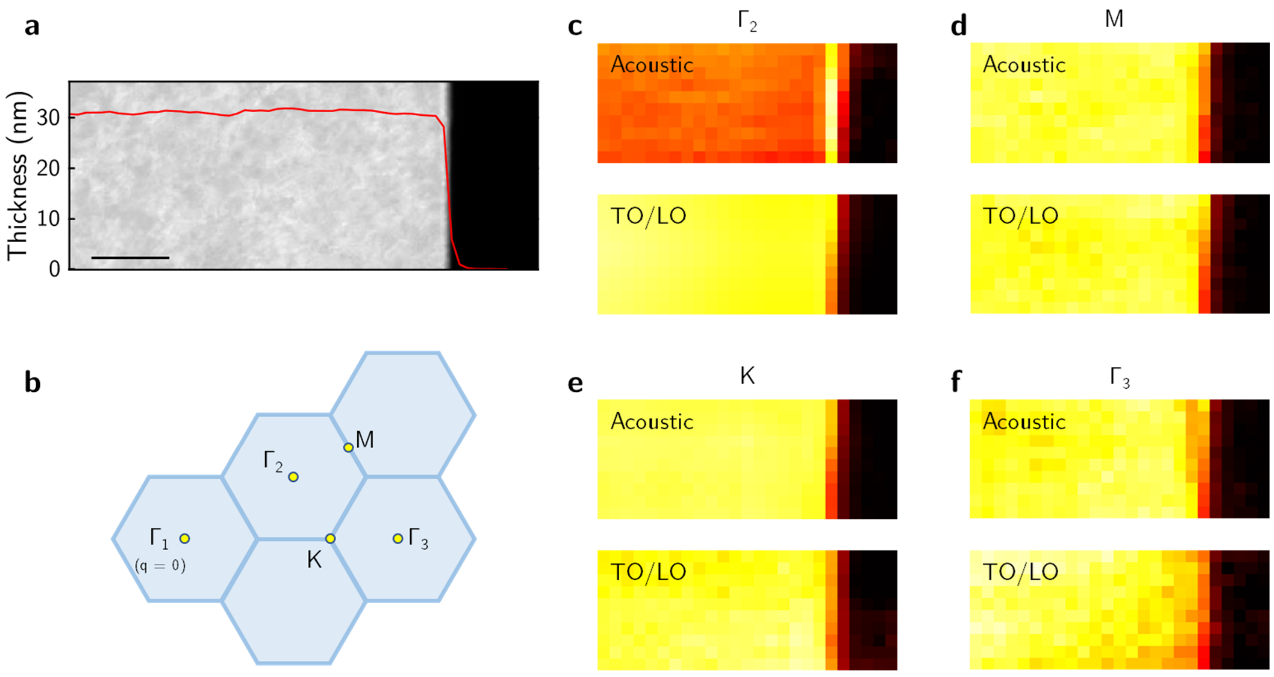

Supplementary Figure 3 4D-EELS measurement on an exfoliated h-BN flake. **a** HAADF image of the h-BN flake under investigation. The red curve superimposed on the image is the thickness line profile calculated by log-ratio technique[1]. Scale bar, 100 nm. **b** High-symmetry points in the momentum space, around which the phonon intensity maps are shown in **c-f**. The first BZ center is denoted by Γ_1. **c-f** Phonon intensity maps at various momentum transfers.

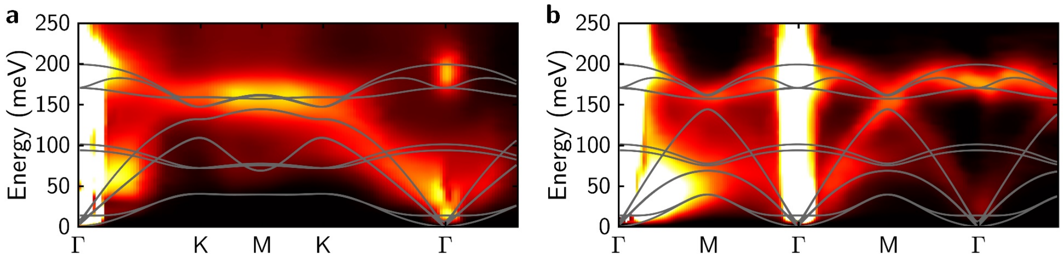

Supplementary Figure 4 Quasi-elastic-line-removed phonon dispersion diagrams of a h-BN flake along high-symmetry lines ΓKMKΓ (**a**) and ΓMΓMΓ (**b**). Solid curves are DFPT calculation for bulk h-BN crystals.

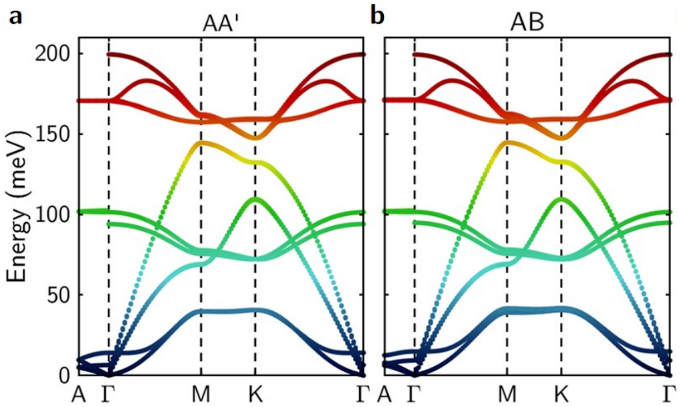

Supplementary Figure 5 Calculated phonon dispersion of bulk BN crystals with AA' (**a**) and AB (**b**) stacking orders. Calculation parameters are listed in Methods.

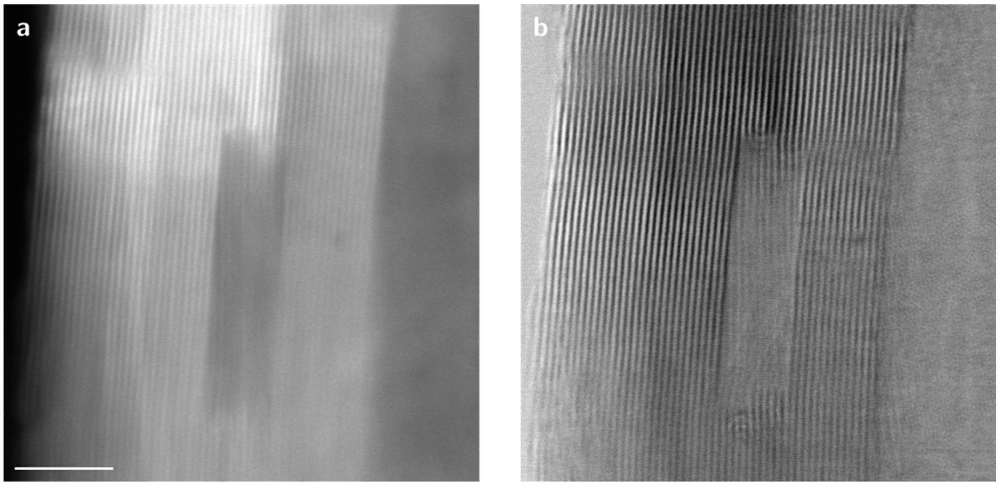

Supplementary Figure 6 Typical void structure in BNNTs. **a** A HAADF image of a typical void in BNNTs, in which atomic fringes are clearly observable. In the void region some constituting shells are missing. Similar voids are common in our samples. Scale bar, 5 nm. **b** Corresponding bright field image.

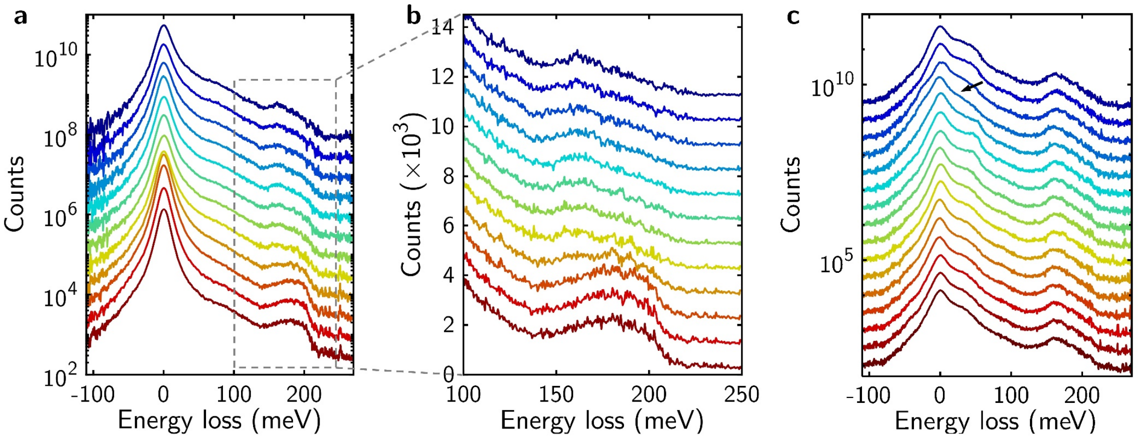

Supplementary Figure 7 EELS line profiles in Figure 4 without elastic-line removal. **a** Raw spectra for Figure 4g in log scale. **b** Zoom-in view of **a** in linear scale to better visualize the energy shift of the TO/LO peak. **c** Raw spectra for Figure 4h in log scale. In **a** and **c**, each line is multiplied by a factor of $\sqrt{10}$ for clarity; in **b** each line is successively shifted upward by 1000 counts. All spectra in this figure are after energy alignment and denoising, but without background-subtraction.

Supplementary Table 1 Experimental parameters in 4D-EELS data acquisition.

	BNNT (Supplementary Movie 1, 2)	h-BN (Supplementary Movie 3, 4)
Beam energy	60 keV	30 keV
Spectral sampling	0.498 meV/channel	0.342 meV/channel
Convergence semi-angle	1.5 mrad	1.5 mrad
Data dimension ($x \times y \times q \times \omega$)	12 × 18 × 800 × 2048	25 × 10 × 800 × 2048
Field of view	153 nm × 230 nm	600 nm × 240 nm
Acquisition time	10 s/pixel, 36 min in total	10 s/pixel, 42 min in total
Energy resolution	15 meV	10 meV
Momentum resolution	0.3 $Å^{-1}$	0.2 $Å^{-1}$
Spatial resolution	4 nm	7 nm

Supplementary References

1 Malis, T., Cheng, S. C. & Egerton, R. F. EELS log-ratio technique for specimen-thickness measurement in the TEM. *J. Electron Microsc. Tech.* **8**, 193-200 (1988).
