# Supplementary material for: Four-dimensional vibrational spectroscopy for nanoscale mapping of phonon dispersion in BN nanotubes
Source: Nat Commun. 2021 Feb 19;12:1179. doi: 10.1038/s41467-021-21452-5 (PMC7896073; doi:10.1038/s41467-021-21452-5)
Supplement: Supplementary file 2 — Description of Additional Supplementary Files [file 41467_2021_21452_MOESM2_ESM.pdf]

Description of Additional Supplementary Files

Supplementary Movie 1

4D-EELS dataset for a BNNT acquired with the slot aperture placed perpendicular to the tube axis (blue rectangle in Fig. 2a-c).

Supplementary Movie 2

4D-EELS dataset for a BNNT acquired with the slot aperture placed at an angle of 60° to the tube axis (green rectangle in Fig. 2a-c).

Supplementary Movie 3

4D-EELS dataset for a h-BN flake acquired with the slot aperture placed along ΓKMKΓ line.

Supplementary Movie 4

4D-EELS dataset for a h-BN flake acquired with the slot aperture placed along ΓMΓMΓ line.
